# Evaluation of the Endo-Uro trainer for semi-rigid ureteroscopy training

**DOI:** 10.1177/1756287219875584

**Published:** 2019-09-22

**Authors:** Sharanya Palaneer, Abdullatif Aydin, Hasaneen Al Janabi, Ahmed Al-Jabir, Nicola Macchione, Muhammad Shamim Khan, Prokar Dasgupta, Kamran Ahmed

**Affiliations:** MRC Centre for Transplantation, Guy’s Hospital, King’s College London, UK; MRC Centre for Transplantation, 5th Floor Southwark Wing, Guy’s Hospital, King’s College London, SE1 9RT, UK; MRC Centre for Transplantation, Guy’s Hospital, King’s College London, UK; MRC Centre for Transplantation, Guy’s Hospital, King’s College London, UK; ASST Santi Paolo e Carlo, Università degli Studi di Milano, Italy; MRC Centre for Transplantation, Guy’s Hospital, King’s College London, UK; Department of Urology, Guy’s and St. Thomas’ NHS Foundation Trust, London, UK; MRC Centre for Transplantation, Guy’s Hospital, King’s College London, UK; Department of Urology, Guy’s and St. Thomas’ NHS Foundation Trust, London, UK; MRC Centre for Transplantation, Guy’s Hospital, King’s College London, UK; Department of Urology, King’s College Hospital NHS Foundation Trust, London, UK

**Keywords:** education, endo-urology, simulation, training, ureteroscopy

## Abstract

**Background:**

The aim of this study was to evaluate the validity of evidence of the Endo-Uro Trainer (SAMED, Dresden, Germany) for semi-rigid ureteroscopy.

**Methods:**

Novice (*n* = 29), intermediate-level (*n* = 25), and expert (*n* = 24) urological surgeons were recruited to participate in the study. Novices were allocated randomly to Groups A and B, where A performed two set procedures using the already validated Uro-Scopic Trainer (Limbs and Things, Bristol, UK), and Group B used the Endo-Uro trainer. Subsequently they were crossed over to perform the same two procedures using the other model. Intermediate and expert groups performed the same procedure on the Endo-Uro trainer only. Objective Structured Assessment of Technical Skills (OSATS) and the procedural times were collected and analyzed. All participants were invited to complete a final evaluation survey.

**Results:**

The evaluation survey revealed a realism rating in all aspects, with a mean Likert rating of 4.04/5. Significant differences were observed in performance time between novices and experts (*p* = 0.0014), and between intermediates and experts (*p* = 0.0113). OSATS scores differed significantly between all groups (*p* < 0.0001). Group B novices showed statistically significant improvement in performance time (*p* = 0.0012) and OSATS scores (*p* = 0.0439) after the crossover. Significant differences in performance time (*p* = 0.0025) between groups A and B were also observed post-crossover.

**Conclusions:**

This study demonstrated content validity for the Endo-Uro Trainer model. In addition, the model was shown to be capable of differentiating levels of experience, which contributes to the acceptance of the validity hypothesis. Improvement in performance using the model demonstrates its effectiveness for training.

## Introduction

With rising challenges in surgical training, especially the limited number of hours available for teaching, simulation-based skills acquisition has gained significant popularity to supplement the operating room (OR) experience. In addition to teaching basic skills, it can be used to simulate complex scenarios and procedural complications, including potential errors made by the surgeon.^1^

Several types of simulators are available, including virtual reality (VR) and live animal models/human cadavers. Bench-top models are stand-alone simulation models that can be entirely synthetic or can incorporate animal tissue. They are relatively cheap compared with other types of simulation, and are reusable, with procedures able to be repeated multiple times in a single model. This also makes them suitable for assessment purposes.^2^

This prospective study aimed to assess the validity evidence of the Endo-Uro Trainer (SAMED, Dresden, Germany), a high-fidelity endo-urological simulator, for training for semi-rigid ureteroscopy.^3^ In addition, a secondary aim was to analyze the learning curve amongst novices for semi-rigid ureteroscopy.

## Methodology

This study invited novice medical students, junior trainees, and consultant specialists, and was comprised of two parts: a randomized controlled trial (RCT); and a prospective, observational, and comparative study between different levels of participants.

### Training models

**Figure 1.**
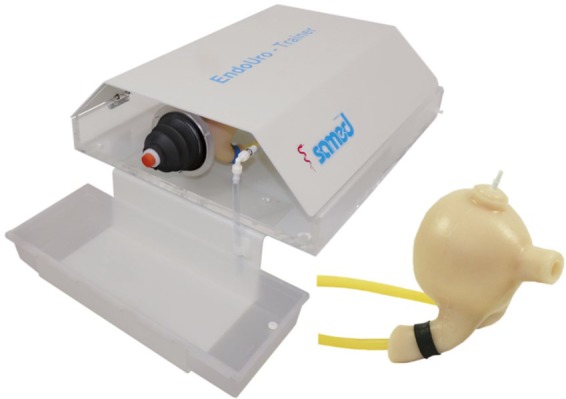
The Endo-Uro trainer (Samed, Dresden, Germany).

Endo-Uro Trainer (SAMED, Dresden, Germany) is a high-fidelity bench model that can be used to simulate cystoscopy, semi-rigid ureteroscopy and flexible uretero-renoscopy (Figure 1). It consists of a bladder, with trigone and two ureteric orifices; two ureters and two kidney models with a renal pelvis and calyces. In addition, it consists of a stenosis unit for ureteric stenosis, and an irrigation pump. The pump can be connected to the scope, which allows irrigation during the procedure. The drainage system uses plastic tubes connected to the model to remove excess water. The kidney units are held together using elastic bands. The ureters and the bladder are easily detachable. The model can be charged using prepared calculi into the location of choice.

A previously validated simulator,^4^ the Uro-Scopic Trainer (Limbs and Things, Bristol, UK), was used as a control. This model consists of a rigid pelvis containing a urethra, bladder, two ureters, and two kidneys with renal pelvises, as well as an irrigation mechanism allowing drainage.

### Study design

All participants (*n* = 78) were required to perform standardized semi-rigid ureteroscopy and mid-ureteric stone basketing tasks for a hypothetical case of a patient with a right mid-ureteric stone. Participants were stratified by procedural experience, with novices defined as medical students with no previous experience in ureteroscopy. Trainee urologists who had performed <150 semi-rigid ureteroscopies were considered as intermediates. Consultants and trainees who had performed >150 semi-rigid ureteroscopies were deemed as part of the expert group.

### RCT component

**Figure 2.**
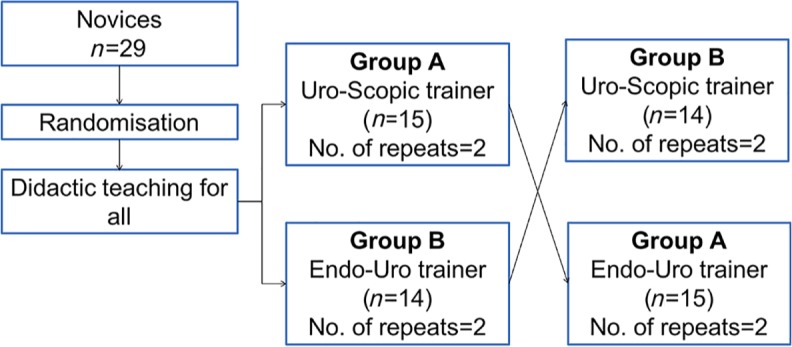
Study-design flowchart for the randomized controlled trial component.

A prospective, randomized parallel controlled trial (Figure 2) was performed in the novice cohort (*n* = 29) with two arms: Group A (*n* = 15), who began on the Uro-Scopic trainer; and Group B (*n* = 14), who began training with the Endo-Uro trainer. Participants were randomized using an online randomization tool (www.randomizer.org).

All the novices were introduced to the procedure using the same presentations and demonstrations by faculty. Following this, participants were asked to perform the procedure on their allocated simulator. The groups were then crossed over and Group A performed the procedure using the Endo-Uro Trainer model and Group B used the Uro-Scopic trainer. Each participant performed the procedure twice in the allocated model.

### Observational study

The intermediate and expert groups (*n* = 49) each performed the procedure once using the Endo-Uro Trainer only. Upon completion of the tasks (in both parts of the study), all participants were given a structured questionnaire, which was used to collect demographic information and to assess realism/usefulness. The participants were asked to score the realism of the model. Trainees and experts were asked to score how well the model performed for six different skills: cystoscopy and urethral examination, bladder examination, anatomical identification, guidewire insertion, semi-rigid ureteroscope insertion and navigation, and stone extraction. This was done using a 5-point Likert Scale with a score of 3/5 determined as an acceptability threshold. In addition, trainees and experts were asked to assess the feasibility and acceptability of the simulator as a training tool alongside other questions related to educational value.

### Outcome measures and evaluation

Outcome measures were procedural time and skills performance (global rating score). All procedures were recorded, and timings were recorded using the procedural videos. The start of the procedure was defined as the point of entry of the cystoscope into the urethral opening. The end was defined as the complete removal of the semi-rigid ureteroscope, along with the basket and the stone. The videos were analysed by an expert urological surgeon and were scored using the Objective Structured Assessment of Technical Skills (OSATS) tool.

### Statistical analysis

RCT data was analyzed on a per-protocol basis. All the data was recorded using Microsoft Excel (Microsoft Corporation, Redmond, WA, USA). The statistical tests were performed using GraphPad Prism 7.04 (Prism, La Jolla, CA, USA) data analysis software. To evaluate the validation hypothesis, relations to others was assessed using a two-tailed Mann–Whitney *U* test was performed to identify significant differences in performance between procedural experience levels. Group B participants’ per-formances, before and after crossover, were compared using a paired *t* test to compare performance of the simulator to a current well-validated simulator. An unpaired *t* test was performed between Group A and Group B participants before and after crossover. The learning curve was analyzed using ANOVA tests. All values with a *p* < 0.05 were considered statistically significant.

### Ethics

This study was performed in accordance with the Declaration of Helsinki and the ethical standards of the institutional research committee. Participants were informed of the aim of this exploration, and their right to decline to participate, and their right to withdraw from the study at any time. All participants had given prior written consent to study participation and data publication. No formal ethical approval was sought for this exploration of training effect, since significant social, emotional, physical, legal, or financial ethical risks were not identified or anticipated.

## Results

### Participant demographics

The novice group consisted of 29 medical students from UK Medical schools, with an average age of 20.8 years. Of the 49 specialists and trainees included in the study, 25 were intermediates and 24 were experts; 55 were male with a mean age of 37.2 years (±9.1). The participants in the intermediate group ranged from 1 to 6 years of urology specialist training and 4–150 semi-rigid ureteroscopies performed. In the expert group, 2 were trainees who had performed >150 semi-rigid ureteroscopies, and 22 were attending urologists. Of the non-novice participants, 29 said that they have had previous experience of using surgical simulators, and 20 had no experience.

### Realism and content assessment

Participants were asked to assess the realism of the model. The overall mean realism score for all the tasks was 4.04/5 (±0.17) on a 5-point Likert scale (1 = unrealistic, 5 = very realistic). ‘Semi-rigid ureteroscope insertion and navigation’ achieved the highest score (4.23 ± 0.79), whereas ‘Bladder examination’ was rated as the least realistic (3.87 ± 0.82). However, all components scored >3/5.

The Intermediate and Expert groups (*n* = 49) were asked to score the model on how well it allowed the performance of each task. Semi-rigid ureteroscope insertion and navigation once again scored the highest (4.17 ± 0.78), with guidewire insertion (4.0 ± 0.92) scoring the least. Participants agreed that the Endo-Uro Trainer is a realistic training tool (4.22 ± 0.63), the model is a good way to learn procedural steps (4.40 ± 0.63), and that it must be used routinely for training and assessment (4.05 ± 0.85).

### Relation with other variables

**Figure 3.**
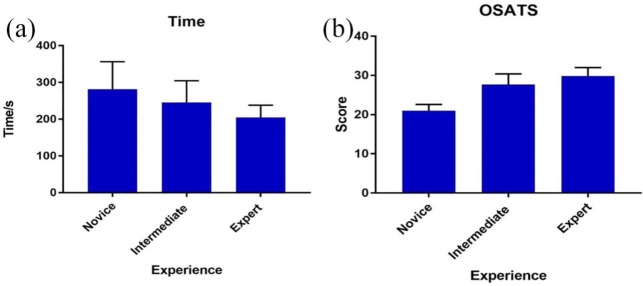
(a) Overall performance time; (b) OSATS score. OSATS, Objective Structured Assessment of Technical Skills.

OSATS scores and performance times of Novice, Intermediate, and Expert groups were compared (Figure 3a,b). There was a significant difference in performance times between Novices and Experts (*p* = 0.0014; Table 1) and Intermediates and Experts (*p* = 0.0113). However, the difference between Novices and Intermediates was not statistically significant (mean 281.4 ± 74.9 *versus* 261.5 ± 103.5, *p* = 0.2135). Statistically significant differences were seen in OSATS scores (Table 1) between Intermediate and Novices (*p* < 0.0001), Intermediate and Experts (*p* = 0.0001), and Novices and Experts (*p* < 0.0001).

**Table 1.** Mean performance time and OSATS scores comparisons between participant groups.

Group	Test	Performance time (s)	*p* value	Mean OSATS score	*p* value
Novice *versus* Expert	Mann–Whitney *U*	281.4 ± 74.9 *versus* 206.7 ± 36.4	*p* = 0.0014	21.0 ± 1.6 *versus* 30.1 ± 2.1	*p* < 0.0001
Novice *versus* Intermediate	Mann–Whitney *U*	281.4 ± 74.9 *versus* 261.5 ± 103.5	*p* = 0.2135	21.0 ± 1.6 *versus* 26.9 ± 3.1	*p* < 0.0001
Intermediate *versus* Expert	Mann–Whitney *U*	261.5 ± 103.5 *versus* 206.7 ± 36.4	*p* = 0.0113	26.9 ± 3.1 *versus* 30.1 ± 2.1	*p* = 0.0001
Novice groups					
Group A *versus* B (pre-crossover)	*t* test (Unpaired)	268 ± 61.9 *versus* 281 ± 72.2	*p* = 0.9807	20.2 ± 1.1 *versus* 21 ± 1.5	*p* = 0.1538
Group A *versus* B (post-crossover)	*t* test (Unpaired)	246.9 (±55.4) *versus* 223.7 (±42.6)	*p* = 0.0025	20.6 ± 1.69 *versus* 22.1 ± 1.2	*p* = 0.0984
Group B pre- *versus* post-crossover	*t* test (Paired)	281.4 ± 72.2 *versus* 223.7 ± 42.6	*p* = 0.0012	21 ± 1.5 *versus* 22.1 ± 1.2	*p* = 0.0439

OSATS, Objective Structured Assessment of Technical Skills.

### Randomized controlled trial

The results of the first trial were analyzed to identify differences in baseline skill levels between the groups. An unpaired *t* test showed no significant difference in means between the two groups for performance times (*p* = 0.9807) and OSATS score (*p* = 0.1538).

Post crossover trials were used to compare the two models as training tools. Performance time was significantly lower in Group B participants, with a mean difference of 94.78 ± 28.44 s (*p* = 0.0025). However, difference in OSATS scores was statistically insignificant (*p* = 0.0984).

A paired *t* test demonstrated significant improvement in the Group B performance time (*p* = 0.0012) and OSATS scores (*p* = 0.0439) post-crossover.

### Learning curve analysis

One-way ANOVA of performance times, and OSATS scores from all four trials, showed statistically significant differences (*p* < 0.0001). A paired *t* test between trial 1 and trial 4 showed improvement in timings, with a difference in mean of 137.1 ± 151.4 s (*p* < 0.0001). However, the performance between trial 2 and 3 plateaued, and the difference was statistically insignificant (*p* = 0.9534). Similarly, for OSATS, the ANOVA was statistically significant (*p* < 0.0001). Difference in mean scores between trials 1 and 4 was 5.048 ± 3.457 (*p* < 0.0001), with the trial 4 score being higher. Comparison between trials 2 and 3 showed that performance in trial 2 was better than trial 3, with a mean difference of 3.286 ± 2.348 (*p* < 0.0001).

## Discussion

Simulation-based surgical education has gained considerable popularity over the years, and several models have been introduced for urolithiasis training. The Uro-Mentor (Simbionix, Lod, Israel), a high-fidelity virtual reality simulator, is the most thoroughly validated model, and, unlike other models, it has demonstrated all domains of validity for ureteroscopy training.^5,6^ The Uro-Scopic Trainer (Limbs & Things, Bristol, UK) is the most commonly used bench model, and allows rigid and flexible ureteroscopy training.^6^ It has demonstrated face, construct, and concurrent validity,^4,7–9^ as per the old framework of validity.

Validity is used to evaluate if a simulator is effective in simulating the desired task. It is an ongoing process, with a validity hypothesis needing evidence to accept or deny it. As per Noureldin and colleagues,^3^ this evidence can consist of many elements, including its content (i.e. whether the simulators procedures/physical design is adequate to simulate the desired target procedure) as well as whether there is a relationship between assessment scores produced using said simulator, and whether they follow a theoretical relationship with other variables (such as procedural experience).

The primary outcome of this study was the validation of the Endo-Uro Trainer for semi-rigid ureteroscopy training. Parallel use of the Uro-Scopic trainer in the novice group allowed comparison between the two models. In addition, multiple repetitions of the procedure by each novice helped to analyze learning curves. The data from questionnaires showed that the Endo-Uro trainer was considered a realistic simulator for semi-rigid ureteroscopy. For content validation, the expert and intermediate groups scored the model ⩾4/5 for performance of each task. The questionnaire also demonstrated that the most common weaknesses of the Endo-Uro Trainer identified was the lack of a distinct urethra. In contrast, the Uro Scopic Trainer has a prosthetic penis with a 10 cm urethral component, which allows urethroscopy simulation, whereas the Endo-Uro Trainer has only a 5 cm passage between the inlet and the bladder. Therefore, simulation of urethral navigation and examination may be difficult in the latter.

The performances of the Novice, Intermediate, and Expert groups on the Endo-Uro Trainer were compared to demonstrate a relationship with an external variable. Significant differences were observed (Table 1), with the intermediate group obtaining better OSATS scores than novices, and experts performing best for both measures. This suggests that the model is able to differentiate various levels of expertise. Overall, the difference in OSATS was more significant than the performance time.

The trial with the novices was performed to assess the Endo-Uro Trainer as a training tool, and compare it with the Uro-Scopic Trainer. Pre-crossover trials were considered as the training stage, and post-crossover trials were to assess participants’ skills. The use of a different model in the assessment allowed identification of transferrable skills gained from previous trials. Similar results between the groups in the pre-crossover trials showed similar skill levels. Significantly improved performance among Group B participants, with reduced timing and higher OSATS scores in trial 3 with the Uro-Scopic trainer, compared with the first trial with the Endo-Uro trainer, contributes to the acceptance of the validity hypothesis. Comparison of post-crossover results between Group A and Group B showed significantly better performance times in Group B. However, OSATS scores failed to show statistically significant differences. Therefore, further tests with larger cohorts are required to produce stronger comparisons of the two models.

The secondary aim of the study was to identify the learning curve for novices. The change in performance and the gradient of the learning were analyzed over four trials. There was a rapid improvement in performance between trial 1 and trial 2, and a similar gradient between trial 3 and trial 4. However, there was a plateau in the gradient between trial 2 and trial 3. This was attributed to the switch in the model used between these trials, which could have had a negative impact on performance, as participants were required to re-adjust to a new setting.

Mishra and colleagues analyzed the change in performance, among medical students, over three repetitions of a procedure.^9^ Rate of skills acquisition was identified as a cause for the change in performance between the trials. Early stages were associated with rapid skill acquisition, displayed as a steep gradient in performance. However, with repetitions, skill acquisition slows, resulting in a plateau.^9^

This study demonstrates validity evidence for the Endo-Uro Trainer as comparable to the Uro-Scopic Trainer, which is the most thoroughly evaluated bench model. Brunckhorst and colleagues utilized the Uro-Scopic trainer in a training curriculum combining technical and nontechnical skills training.^10^ A RCT involving 32 medical students showed significantly better performance in the group trained using the curriculum. Content validity, acceptability, and feasibility were also demonstrated in the study.^10^ Therefore, a future research goal would be to incorporate this model into training curricula.

Several types of simulators have been introduced, with specificities to a wide range of urological procedures.^11,12^ The Endo-Uro Trainer is a high-fidelity bench model for ureteroscopy training. The study was able to demonstrate the content validity for the model. In addition, the model was shown to be capable of differentiating novices from intermediates and experts, which contributes to the acceptance of the validity hypothesis. It improved the performance of novices trained using the model, showing that it is an effective training tool for the procedure.
